# Noninvasive cardiac output and blood pressure monitoring cannot replace an invasive monitoring system in critically ill patients

**DOI:** 10.1186/1471-2253-9-6

**Published:** 2009-10-12

**Authors:** John F Stover, Reto Stocker, Renato Lenherr, Thomas A Neff, Silvia R Cottini, Bernhard Zoller, Markus Béchir

**Affiliations:** 1 Surgical Intensive Care Unit, University Hospital of Zurich, CH 8091 Zurich, Switzerland

## Abstract

**Background:**

Monitoring of cardiac output and blood pressure are standard procedures in critical care medicine. Traditionally, invasive techniques like pulmonary artery catheter (PAC) and arterial catheters are widely used. Invasiveness bears many risks of deleterious complications. Therefore, a noninvasive reliable cardiac output (CO) and blood pressure monitoring system could improve the safety of cardiac monitoring. The aim of the present study was to compare a noninvasive versus a standard invasive cardiovascular monitoring system.

**Methods:**

Nexfin HD is a continuous noninvasive blood pressure and cardiac output monitor system and is based on the development of the pulsatile unloading of the finger arterial walls using an inflatable finger cuff. During continuous BP measurement CO is calculated. We included 10 patients with standard invasive cardiac monitoring system (pulmonary artery catheter and arterial catheter) comparing invasively obtained data to the data collected noninvasively using the Nexfin HD.

**Results:**

Correlation between mean arterial pressure measured with the standard arterial monitoring system and the Nexfin HD was r^2 ^= 0.67 with a bias of -2 mmHg and two standard deviations of ± 16 mmHg. Correlation between CO derived from PAC and the Nexfin HD was r^2 ^= 0.83 with a bias of 0.23 l/min and two standard deviations of ± 2.1 l/min; the percentage error was 29%.

**Conclusion:**

Although the noninvasive CO measurement appears promising, the noninvasive blood pressure assessment is clearly less reliable than the invasively measured blood pressure. Therefore, according to the present data application of the Nexfin HD monitoring system in the ICU cannot be recommended generally. Whether such a tool might be reliable in certain critically ill patients remains to be determined.

## Background

Cardiovascular monitoring is a standard procedure in critical care medicine. Traditionally invasive techniques like pulmonary artery catheter (PAC) and peripheral artery catheters are widely used [1-3]. A further possibility of cardiovascular monitoring is the PiCCO system[4]. These techniques are well established and validated [5-7]. Besides the advantages of these invasive techniques for clinical decision-making, these methods both bear the risks of deleterious complications as e.g., bleeding, pneumothorax and infection; PAC is also associated with the risk of inducing pulmonary artery rupture [8,9]. Furthermore, these systems are cost intensive. Nexfin HD is a continuous noninvasive blood pressure (BP) and cardiac output (CO) monitor (former called Modelflow, Finapress) [10]. Its advantages are the noninvasive assessment and its very easy application within minutes [11]. This device has been successfully used in healthy volunteers during orthostasis or for optimization of cardiac resynchronization therapy[12,13]. To date, however, we still lack data regarding the applicability of this system in an intensive care unit (ICU) setting. The present study was designed to evaluate the Nexfin HD device under clinical conditions in a surgical ICU. Our hypothesis was that the Nexfin device produces results comparable to a standard invasive blood pressure monitoring system, i.e., PAC and arterial line. The acceptance of a new method should be judged against the ± 10-20% accuracy of the current reference method. Consequently, the limits of agreement between the new and the reference technique of ± 30% is considered acceptable [14].

## Methods

Following approval by the local Ethics Committee which waived the need for written informed consent for this retrospective data analysis, patient data from a total of 10 patients treated on our intensive care unit in 2007 were analyzed.

### Nexfin HD technique

The method of the Nexfin HD (BMEYE B.V, Amsterdam, Netherlands) is based on the development of the pulsatile unloading of the finger arterial walls using an inflatable finger cuff with a built-in photoelectric plethysmograph. While continuously measuring BP the monitor calculates CO. The method is described in detail by de Wilde and colleagues [15]. The monitoring system is an approved medical device in Switzerland.

### Patients and data analysis

Data of ten critically ill patients in need of cardiovascular monitoring with PAC (Edwards life sciences Germany GMBH, Unterschleissheim, Germany) and invasive blood pressure measurement (Drägger monitoring systems, Carbamed, Bern, Switzerland) in which the Nexfin HD was additionally used during the stay in the ICU were collected. Hourly Nexfin HD measurements obtained during an 8 hour period were compared to arterial blood pressure values and CO, respectively. A total of 80 data points for blood pressure and CO were included.

### Application of monitoring technique

Apart from PAC monitoring which was used in a standardized fashion [1-3], the Nexfin HD device was installed by applying a compact and simple cuff on the middle finger according to the manufacturers' recommendations. Signals derived from the cuff were analysed and presented in real time on the Nexfin HD stand alone device.

### Statistical analysis

The results between the 2 methods were analyzed statistically using correlation and linear regression analysis, including calculation of bias and precision (Bland-Altman analysis) [16] as well as calculation of the percentage error according to Critchley and Critchley [14] (Statview 4.5, abacus concepts). Acceptance of a new technique should rely on limits of agreement of up to ± 30% [14]. Data are presented as mean ± standard deviation.

## Results

### Baseline characteristics and clinical data

A total of ten critically ill patients monitored with PAC, standard invasive arterial blood pressure monitoring and Nexfin HD were included in this study. Arrhythmias were not present during the recording periods. Four patients were lung transplant recipients, 4 patients were liver transplant recipients and 2 suffered from severe ARDS.

Mean age of the patients was 54 ± 12 years, average weight was 74 ± 17 kg, average height was 168 ± 15 cm, average mean arterial pressure (MAP) was 77 ± 13 mmHg, mean heart rate was 98 ± 18 bpm; four of the 10 patients were males.

All patients required norepinephrine as a vasoconstrictor agent for hemodynamic stability. Mean norepinephrine dose during data assessment was 12 ± 12 μg/min (range 2-29 μg/min). Mean SAPS II score was 36 ± 17 and the ICU mortality was 3/10 (30%).

### Comparison of blood pressure measurement

**Figure 1:**
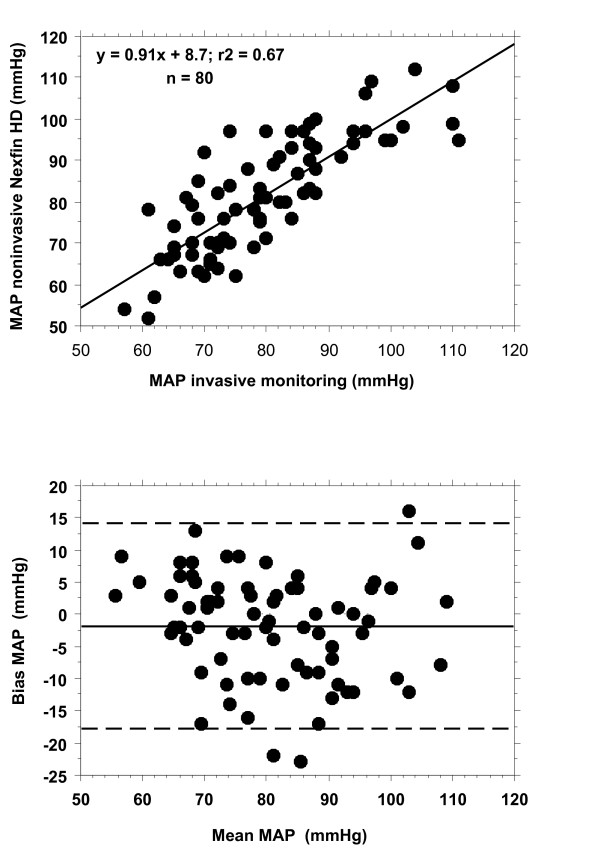
**Blood pressure measurement (black symbols)**. Regression analysis (above) and Bland- Altman analysis (bias and precision plot) (below).

Correlation, regression analysis and the Bland-Altman analysis are shown in Figure 1. Correlation between MAP determined invasively and MAP assessed by Nexfin was r^2 ^= 0.67. Average MAP derived invasively was 80 ± 12 mmHg with a bias of -2 mmHg and 2 standard deviations of ± 16 mmHg.

### Comparison of cardiac output measurement

**Figure 2:**
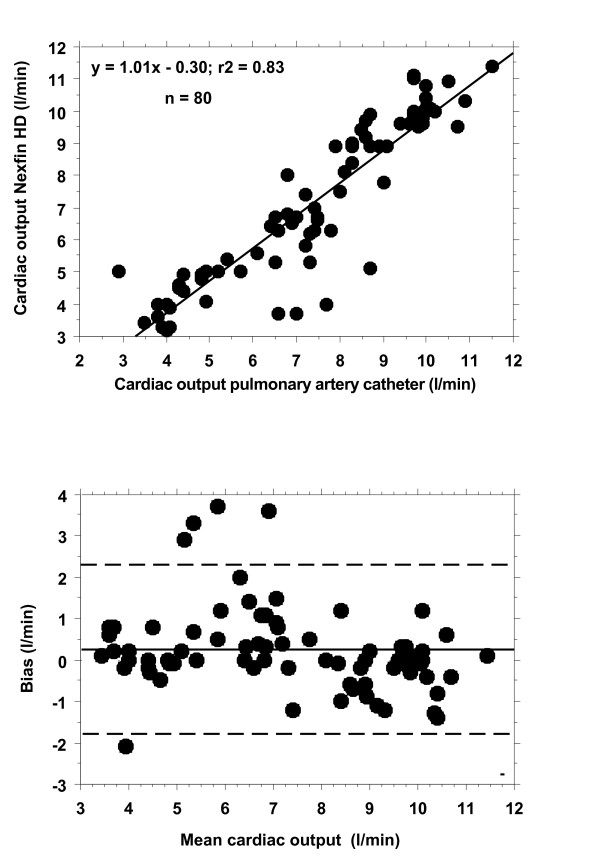
**Cardiac output measurement (black symbols)**. Regression analysis (above) and Bland- Altman analysis (bias and precision plot) (below).

Correlation, regression analysis and the Bland-Altman analysis are shown in Figure 2. Correlation between CO derived by PAC and CO assessed by Nexfin was r^2 ^= 0.83. Mean CO determined by PAC was 7.2 ± 2.3 l/min with a bias of 0.23 l/min, two standard deviations of ± 2.1 l/min and the percentage error was 29%.

## Discussion

The present study revealed that the noninvasive Nexfin HD system cannot substitute standard invasive blood pressure measurement while CO monitoring appears more reliable in the presently investigated group of ten critically ill patients.

The mean difference in MAP between the 2 methods was 2 ± 8 mmHg (2 SD were ± 16 mmHg and r^2 ^= 0.67). This is in contrast to the results reported by Schattenkerk et al, who described a good correlation between the Nexfin HD and blood pressure measurements determined by the Riva-Rocchi/Korotkoff technique (RRK) [17]. Methodological differences preclude a direct comparison of their patients with the presently investigated patients: Schattenkerk et al., compared 2 non-invasive techniques based on cuff measurement without including an invasive method and did not investigate critically ill patients in need of vasopressors.

A possible explanation for the observed difference between the 2 systems might be diminished arterial perfusion in the fingers of our subjects. However, based on the fact that for the same norepinephrine concentration noninvasive blood pressure was higher compared to the invasive blood pressure in approximately 50% of the investigated patients it appears that the finger plethysmography was not to associated with impaired perfusion.

Clinical and therapeutic decision making is weakened by the calculated standard deviation of ± 8 mmHg for the MAP determined by the Nexfin HD device. I.e., for a MAP 65 mmHg, the defined blood pressure target in septic patients [18], we must expect a real MAP of 57 or 73 mmHg. Consequently, the MAP is either too low or too high, thereby resulting in excessive or insufficient therapeutic interventions. This, in turn, might induce additional damage.

Therefore, our comparison of blood pressure measurement indicates that the noninvasive technique cannot properly replace an invasive system.

CO determined non-invasively using the Nexfin HD device was within the recommended limits of agreement, suggesting a fair to good accuracy. However, CO measurement- although very interesting in critically ill patients - does not have the same importance within the therapy of critically ill patients as the MAP. Contrary to MAP CO is not a target value within the early goal directed therapy (EGDT) for septic patients [18]. Thus, although useful, this finding alone does not justify its application in the ICU as a substitute for an invasive monitoring system.

Given the small sample size the results must be interpreted very carefully. Interestingly, the original work of Bland and Altman which describes the statistical method used in this paper [16] was based on a sample size of 17 subjects with a total of 34 data points. Despite a smaller sample size (10 patients) we included twice as many data points (80 data points) in our analysis. Thus, our sample size should allow us to reliably interpret the present results.

Importantly, there were no clinical signs of disturbed microcirculation of the fingers in these patients (discolored and cold fingers) during application of the finger cuff, indicating a safe use of the Nexfin HD system. The noninvasiveness of this technique allows to avoid potential complications related to the invasive nature of the other techniques.

Furthermore, this new finger plethysmography system is very easy to use and quickly to install within minutes and therefore could offer a quick and initial hemodynamic overview, possibly providing important information on trend of MAP and CO. This would allow to bridge the time until a longer lasting invasive monitoring can be installed in the case of a deteriorating patient.

## Conclusion

Although CO measurement seems promising, noninvasive blood pressure assessed by the Nexfin HD device is clearly less reliable than invasive blood pressure measurement. Therefore, according to our data application of the Nexfin HD monitoring system cannot generally be recommended for the ICU. Whether such a tool is reliable in certain groups of critically ill patients has yet to be determined.

## Competing interests

The authors declare that they have no competing interests.

## Authors' contributions

JFS and MB designed the study, performed statistical analysis and drafted parts of the manuscript. RS and SRC collected data and drafted parts of the manuscript. RL and BZ collected the data. TAN assisted in statistical analysis and performed graphs. All authors read and approved the final manuscript.

## Pre-publication history

The pre-publication history for this paper can be accessed here:


